# Quantifying compensatory strategies in adults with and without diagnosed autism

**DOI:** 10.1186/s13229-019-0308-y

**Published:** 2020-02-12

**Authors:** Lucy Anne Livingston, Punit Shah, Victoria Milner, Francesca Happé

**Affiliations:** 1 Social, Genetic and Developmental Psychiatry Centre, Institute of Psychiatry, Psychology and Neuroscience, King’s College London, London, UK; 2 School of Psychology, Cardiff University, Cardiff, UK; 3 Department of Psychology, University of Bath, Bath, UK

**Keywords:** Compensation, Compensatory strategies, Autism, Adaptation, Camouflaging, Social cognition

## Abstract

**Background:**

There is growing recognition that some autistic people engage in ‘compensation’, showing few behavioural symptoms (e.g. neurotypical social skills), despite continuing to experience autism-related cognitive difficulties (e.g. difficulties in social cognition). One way this might be achieved is by individuals consciously employing ‘compensatory strategies’ during everyday social interaction. However, very little is currently known about the broad range of these strategies, their mechanisms and consequences for clinical presentation and diagnosis.

**Methods:**

We aimed to measure compensatory strategies in autism for the first time. Using a novel checklist, we quantified self-reported social compensatory strategies in 117 adults (58 with autism, 59 without autism) and explored the relationships between compensation scores and autism diagnostic status, autistic traits, education level, sex and age at diagnosis.

**Results:**

Higher compensation scores—representing a greater repertoire of compensatory strategies—were associated with having an autism diagnosis, more autistic traits and a higher education level. The link between autism diagnostic status and compensation scores was, however, explained by autistic traits and education level. Compensation scores were unrelated to sex or age at diagnosis.

**Limitations:**

Our sample was self-selected and predominantly comprised of intellectually able females; therefore, our findings may not generalise to the wider autistic population.

**Conclusions:**

Together, our findings suggest that many intellectually able adults, with and without a clinical diagnosis of autism, report using compensatory strategies to modify their social behaviour. We discuss the clinical utility of measuring self-reported compensation (e.g., using our checklist), with important implications for the accurate diagnosis and management of autism and related conditions.

## Background

It is increasingly recognised that a subgroup of people diagnosed with autism spectrum disorder (ASD) can, in certain contexts, appear neurotypical, demonstrating few atypical behaviours. These individuals may show good eye contact, appropriate social reciprocity and no obvious restricted interests [1–3]. Whilst it has been argued that this neurotypical presentation is driven by remediation of cognitive difficulties [4] (i.e. ‘recovery’), there is growing evidence to suggest that neurotypically presenting autistic people continue being autistic at the cognitive level [1, 5]. Drawing on the concept of compensation from neurology (e.g. alternative/adaptive neural processing following brain injury), this recently led to the ‘compensation hypothesis’ [1]. This posits that some people with neurodevelopmental conditions, such as ASD, can compensate for their cognitive difficulties (e.g. in social cognition), using alternative neural routes and psychological strategies to demonstrate neurotypical behaviour (e.g. good social skills). These processes may operate at both conscious and subconscious levels. Compensation in ASD is a topic of rapidly growing interest. It helps, in theory, to explain why some autistic people have apparently better outcomes than others, but equally—given the reliance of diagnosis on observable behaviour—why they may receive a late first diagnosis in adulthood [1, 5, 6], particularly females who are thought to compensate more than males [1, 2, 7–10].

### Approaches to studying compensation in autism

Despite substantial interest in the concept and clinical relevance of compensation in ASD and other neurodevelopmental conditions [11, 12], there is limited empirical work on the topic. Generally speaking, research on ASD has taken two approaches thus far. One approach—the behaviour-cognition discrepancy approach—operationalises compensation as the mismatch between observable behaviour and underlying cognition; that is to say, autistic ‘compensators’ should appear more neurotypical in behaviour than their cognitive profile would otherwise suggest. Accordingly, a handful of studies [2, 3, 13] have quantified social compensatory ability in ASD as the discrepancy between observer-rated social skills and performance on social-cognitive tasks (e.g. measuring theory of the mind—the ability to understand other minds [14]). This approach is advantageous in that it captures the overall output of compensation, both in conscious and unconscious forms, in a fairly objective manner. However, it does not shed light on unsuccessful compensation, that is, strategies that do not necessarily translate to more neurotypical behaviour.

Therefore, a second approach—the self-report approach—has been used to measure the propensity to compensate, through qualitative studies and questionnaires that directly ask autistic people about their experiences using compensatory strategies. Hull and colleagues developed the first such measure, the Camouflaging Autistic Traits Questionnaire (CAT-Q), based on qualitative work with diagnosed autistic adults [15]. The CAT-Q was originally designed to measure camouflaging, which Hull and colleagues defined as the attempt to hide or disguise one’s autistic features. They found that the CAT-Q had distinct ‘masking’ and ‘compensation’ components, the former of which reflects simple, fairly passive strategies to blend in or hide autistic behaviour, whereas the latter reflects active strategies that help individuals to ‘make up’ for social difficulties during social interaction (i.e. appear socially skilled by neurotypical standards). In the present study, we make this same distinction and focus solely on compensation or compensatory strategies.

### Correlates of compensation

Research using these two approaches has helped to advance the concept and establish key correlates of compensation. Compensation in ASD has been linked to better general cognitive abilities, with studies finding that greater social behaviour-cognition discrepancy (i.e. greater compensatory ability) is associated with higher IQ [3] and better executive function [2, 3]. This may reflect the fact that (i) compensatory strategies often involve intellectually derived rules (e.g. when and how long to make eye contact for) and (ii) careful monitoring and switching between strategies is required to compensate successfully. Accordingly, given these links, compensation is proposed to have an adaptive function, supporting autistic individuals to be able to live independently, have successful social relationships and gain and maintain employment [5, 6].

Equally, studies have revealed negative outcomes correlated with compensation. Qualitative research findings suggest that because compensation disguises, but does not necessarily eliminate, autistic difficulties, some individuals may not receive a necessary diagnosis of ASD until adulthood [5–9]. This issue is proposed to be particularly acute for autistic females who compensate to a greater extent than males [1, 2, 7–10]. Delayed diagnosis, for males and females, may consequently delay their access to appropriate clinical support and accommodations in the workplace. Further, studies using both the discrepancy approach and the CAT-Q have found compensation to be linked to poor mental health. This is suggested to be because compensatory efforts are reported as being cognitively demanding, stressful and not always sufficiently successful to ‘pass’ as neurotypical and make social connections with others [1–3, 5, 7, 15, 16].

### Investigating compensatory strategies

Despite important research developments on the correlates of compensation, strikingly little is known about *how* autistic people attempt to compensate in everyday life; that is, the active strategies they use to try to navigate the social world. Although the CAT-Q’s compensation subscale measures some common compensatory strategies (e.g. using scripts in social situations), it does not necessarily capture the full range of strategies, including those used by individuals without a formal autism diagnosis. Furthermore, the strategies measured by the CAT-Q are fairly shallow in nature, involving learning of stringent and context-dependent rules (e.g. copying the gestures of other people). We have previously hypothesised that these may be distinct from deep compensatory strategies, which work flexibly across contexts, because they provide an alternative route to the social-cognitive ability in question (e.g. theory of mind), for example, using complex mental algorithms to predict other people’s thoughts and feelings [1]. This would be akin to a visually impaired person using echolocation; the strategy does not simply circumvent the impairment like a white stick does, but provides an alternative way to form a spatial representation that enables navigation skills. Therefore, in the present study, we aimed to investigate a broader range of strategies ranging from shallow, unsophisticated strategies that only give a superficial impression of neurotypical social skills, to more sophisticated, deep strategies that enable some flexible social understanding.

There are additional issues with studies on compensation so far that we aimed to address in the present study. Overall, there has also been a narrow focus on compensation in diagnosed ASD, without consideration for how the construct aids understanding of social differences more generally. For example, the extent to which individuals without autism (but still experiencing social difficulties) use compensatory strategies is currently unknown. Additionally, it is unclear if people with an autism diagnosis would use more compensatory strategies than non-diagnosed individuals because they potentially have greater social difficulties to compensate for, or fewer strategies, accounting for why they meet diagnostic criteria for ASD in the first place. Therefore, in the present study, we explored compensatory strategies in adults who report social difficulties, regardless of whether they had a formal autism diagnosis. Finally, we note that although qualitative and anecdotal evidence has suggested a link between compensation and later age at diagnosis, no study has to our knowledge directly measured this relationship quantitatively.

### The present study

To address some of these aforementioned issues, we recently conducted a qualitative study that directly and extensively investigated compensatory strategies in adults—with and without an autism diagnosis—who experience social difficulties [5]. Participants were asked to describe qualitatively all the possible strategies they use to overcome difficulties in social situations. This study, providing rich data on autistic people’s lived experiences, confirmed that at least a subgroup of autistic people are able to describe at length their compensatory strategies. Additionally, qualitative analyses highlighted various meaningful types of strategy [5], including masking, shallow compensation and deep compensation. Additionally, we identified an additional strategy type termed ‘accommodation’, which reflects strategies that involve actively seeking environments/people that accommodate one’s cognitive difficulties and strengths. However, due to a lack of quantitative analyses in this study, it was unclear if compensatory strategies (i) significantly differed between people with and without diagnosed autism and (ii) were statistically associated with factors theoretically linked to compensation (e.g. IQ, delayed diagnosis, female sex). Therefore, in the present study, we quantified self-reported (social) compensatory strategies in autism for the first time. By coding participants’ free-text descriptions with a novel 31-item *Compensation Checklist*, quantitative compensation scores were created. Following this, we explored relationships with diagnostic status, autistic traits, highest education level (as a proxy for IQ), age at diagnosis and sex.

We hypothesised that having an autism diagnosis, more autistic traits and a higher education level would be linked to greater self-reported compensation scores. Additionally, as compensation is theorised to delay diagnosis [1, 5, 6] and be central to the female autism phenotype [1, 2], we predicted that older age at diagnosis and female sex would also be associated with higher compensation scores.

## Methods

### Participants

Participants formed a convenience sample of 117 adults (95 females) aged 18–77 years old (*M* = 34.85, *SD* = 13.28), who responded to an advert seeking individuals who use strategies to overcome difficulties in social situations. The advert made explicit that this may include, but was not limited to, individuals with autism. In our sample, 58 participants had an autism diagnosis (‘Diagnosed’) and 59 participants neither had an autism diagnosis nor reported being autistic (‘Non-diagnosed’). Diagnosed participants confirmed their diagnosis [Asperger syndrome (*n* = 33), autism spectrum disorder (*n* = 20), atypical autism (*n* = 2), pervasive developmental disorder-not otherwise specified (*n* = 3)] and the healthcare professional(s) who made the diagnosis. Nineteen additional participants were recruited, who self-identified as autistic but did not have an autism diagnosis; these participants contributed data elsewhere [5], but their data are not included in the current study.

### Materials and procedure

Participants accessed the study online. They answered numerous open-ended questions about their use of social compensatory strategies (see [5] for full methodological details) using free-text response boxes. They also self-reported autistic traits using the 10-item Autism-Spectrum Quotient (AQ10 [17]) and reported their highest level of education using the International Standard Classification in Education [18], which is often used as an IQ proxy [19]. Finally, participants reported their sex at birth, age, whether or not they had a family member with diagnosed autism and, for diagnosed participants only, their age at diagnosis.

### Data coding and analysis

Previous thematic analysis of participants’ text responses identified 31 strategies, which could be conceptually divided into four strategy types (masking, shallow compensation, deep compensation, accommodation). Characteristics of the various strategy types are detailed in Table 1, and full details of the original thematic analysis can be found elsewhere [5].

**Table 1:** Distinctions between masking, shallow compensation, deep compensation and accommodation strategies, derived from Livingston et al. [5]

Strategy type	Description	Specific examples	Overall characteristics
Masking (6 items)	Strategies that involve regulating (increasing/dampening) pre-existing social behaviours	Hold back your true thoughts and opinions; dress and speak like the group you are trying to blend in with; stand in a conversation but say/do very little	• Not very cognitively demanding/tiring • Can become ‘automatic’ with time • Enable one to ‘blend in’ or ‘go unnoticed’ in group situations or from a far • Do not necessarily support active participation in two-way interaction
Shallow compensation (10 items)	Strategies that enable production of neurotypical behaviour (e.g. social behaviour) without solving the cognitive difficulty/difference in question (e.g. continued theory of mind difficulty)	Enact learned scripts and social rules to guide conversations; make or appear to make ‘appropriate’ eye contact; repeat and rephrase what your interaction partner says to give the impression of being a ‘good listener’	• Fairly cognitively demanding/tiring • Less likely to become ‘automatic’ compared to masking strategies • Enable reciprocal social interaction • Not flexible across contexts, doesn’t always emulate natural social interaction and can ‘break down’ under stress/with constant use
Deep compensation (9 items)	Strategies that enable an alternative route to solve the cognitive difficulty in question (e.g. successfully solve theory of mind, albeit differently to neurotypical people)	Flexibly use built catalogue of possible interpretations of others’ mental states, based on a combination of multiple sources of information (e.g. logic, context, facial expression, tone of voice); substitute others’ values/interests with your own or those of a TV/book character to infer their mental state	• Can initially be challenging to devise • Can become ‘automatic’ with time • More flexible than shallow strategies • Support genuine improvements in social cognition (e.g. theory of mind)
Accommodation (6 items)	Strategies that help accommodate, but do not necessarily alter, one’s cognitive difficulty/difference	Work in an environment where your social differences are actively accommodated; live in a foreign country so that your social differences are attributed to ‘being foreign’ by others	• May enable ‘good outcome’ (e.g. employment, good mental health) without autistic behaviour necessarily reducing • May require additional support structures (e.g. family, financial resources) • Can work alongside compensatory strategies, but are ultimately distinct

In the present study, we used the same dataset to quantify self-reported compensatory strategies. We created the 31-item Compensation Checklist using the strategies previously identified (see Additional file 1: Appendix 1). Three raters (LAL, PS, VM) independently coded participants’ text responses for the presence/absence (1/0) of each strategy, blind to diagnostic status (inter-rater reliability: percentage agreement = 87%, Gwet’s AC1 = 0.83 [95% CIs 0.81–0.84]^1^). The four compensation types (masking, shallow compensation, deep compensation, accommodation; see Table 1) were measured separately and summed to create an overall compensation score (possible range 0–31). Higher scores indexed more strategies reported, and therefore a greater self-reported compensation repertoire. An exploratory analysis of unidimensionality and internal congeneric reliability [22] suggested that, although individual strategies within the four different strategy types were not correlated with each other (average inter-item correlation: masking, *r* = .01; shallow compensation, *r* = .06; deep compensation, *r* = .06; accommodation, *r* = .02), the Compensation Checklist has one underlying construct, i.e. compensation (greatest lower bound = 0.82).

Correlations were conducted to explore (i) inter-relationships between various strategy types and (ii) links between compensation scores and diagnostic status, AQ10, education level, age at diagnosis and sex. Variables demonstrating significant relationships with compensation scores were subject to multiple linear regression, to assess their unique ability to predict compensation, whilst statistically controlling for the other related variables. As the strategy types had differing numbers of items and may therefore have unequal weighting in analyses, all analyses were conducted using standardised scores as well as raw scores. To create standardised scores, each strategy score was calculated as a function of the total possible score for that particular strategy type (masking, 6; shallow compensation, 10; deep compensation, 9; accommodation, 6) and summed to form standardised overall compensation scores. Analyses using raw and standardised scores produced a similar pattern of results; therefore, analyses using raw data only are reported. The equivalent analyses using standardised scores can be found in Additional file 1.

## Results

**Fig. 1:**
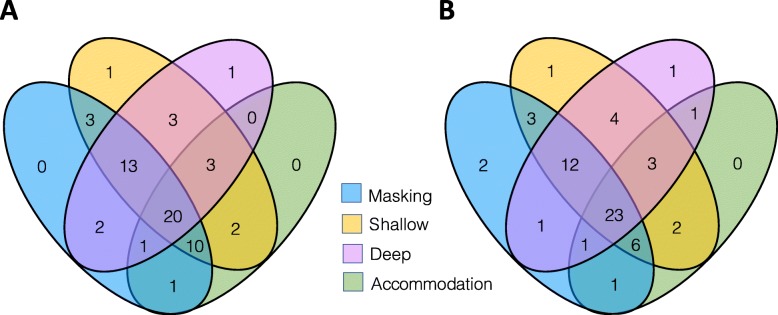
Venn diagrams showing the number of **a** Diagnosed and **b** Non-diagnosed participants that reported using masking, shallow compensation, deep compensation and/or accommodation strategies. Overall, participants were more likely to report strategies across multiple types, than a single strategy type. This pattern was broadly similar between the two groups, but there was a significant group difference in shallow compensation (see Table 4)

Group characteristics are shown in Table 2. Diagnosed and Non-diagnosed groups did not differ significantly in terms of age, sex or education level, but Diagnosed participants demonstrated greater autistic traits (AQ10 scores), in line with previous research [17]. Diagnosed participants were also significantly more likely to have a relative with an autism diagnosis than Non-diagnosed participants. Figure 1 shows that Diagnosed and Non-diagnosed groups broadly reported a similar pattern of strategy use across the four strategy types; for example, both groups were more likely to report strategies across multiple types than a single type.

**Table 2:** Participant characteristics of the Diagnosed and Non-diagnosed groups

	Diagnosed (*n* = 58)			Non-diagnosed (*n* = 59)			Comparison
	*M*	*SD*	Range	*M*	*SD*	Range	
Age	35.83	11.53	18-70	33.88	14.83	18–77	*t*(115) = − 0.79, *p* = .43, *d* = 0.15
Age at diagnosis	30.14	13.84	3–70	–	–		–
Highest education level (max = 7)	4.66	2.08	0–7	4.68	1.78	1–7	*t*(115) = 0.06, *p* = .95, *d* = 0.01
Autistic traits (max = 10)	8.02	1.92	1–10	4.93	2.29	1–10	*t*(115) = − 7.90, *p* < .001, *d* = 1.46
Sex (*n* male, *n* female)	14, 44	–	–	8, 51	–	–	*χ*^*2*^(1) = 2.14, *p* = .14, *Φ =* 0.14
Family member diagnosed with ASD (*n* yes, *n* no)	19, 39	-	–	8, 51	–	–	*χ*^*2*^(1) = 6.07, *p* = .014, *Φ* = 0.23

Highest education level was used as a proxy IQ measure. Greater scores reflect higher education level/greater autistic traits/more strategies. Effect sizes are reported as Cohen’s *d* (0.2 = small, 0.5 = medium, 0.8 = large) or phi *Φ* (0.1 = small, 0.3 = medium, 0.5 = large)

Correlational analyses, shown in Table 3, revealed that the various strategy types were positively and moderately correlated. Additionally, higher education level, AQ10 scores, and having an autism diagnosis, were associated with greater overall compensation and more specifically, shallow compensation. Masking, accommodation and deep compensation showed no significant links with AQ10, diagnostic status or education level, except for accommodation, which was positively correlated with education level. Compensation scores were not significantly correlated with sex or age at diagnosis. Post hoc *t* tests confirmed that there were no significant sex differences across the various strategy types (all *p*s ≥ .25) and that effect sizes were small (*d*s ≤ 0.28). Group comparisons across strategy scores revealed an identical pattern to the correlational analyses. Diagnosed participants reported greater shallow compensation and overall compensation scores than Non-diagnosed participants, but there were no significant group differences for masking, deep compensation or accommodation (see Table 4).

**Table 3:** Correlational analyses

	1	2	3	4	5
Overall compensation (1)	–	.73***	.59***	.55***	.57***
Shallow compensation (2)		–	.13	.16	.28**
Deep compensation (3)			–	.13	.18
Masking (4)				–	.15
Accommodation (5)					–
Autistic traits	.26**	.41***	.01	.07	.05
Highest education level	.22*	.25**	.02	.09	.18*
Sex (1 = female, 0 = male)^a^	− .04	− .11	.03	.07	− .10
Diagnosis (1 = diagnosed, 0 = non-diagnosed)^a^	.21*	.30**	.13	− .03	.03
Age at diagnosis^b^	.11	.04	− .08	.19	.22

Highest education level was used as a proxy IQ measure. Greater scores reflect higher education level/greater autistic traits/more self-reported strategies. Analyses were computed using both raw and standardised strategy scores (see the “Methods” section). A similar pattern of results was found; therefore, analyses using raw scores are reported (see Additional file 1 for analyses using standardised scores). *p < .05, **p < .01, ***p < .001. ^a^Point-biserial correlations. ^b^Diagnosed group only (*n* = 58)

**Table 4:** Group comparisons of strategy scores

	Diagnosed (*n* = 58)			Non-diagnosed (*n* = 59)			
	*M*	*SD*	Range	*M*	*SD*	Range	Comparison
Overall score (max = 31)	6.81	3.32	1–16	5.56	2.56	1–13	*t*(115) = −2.29, *p* = .024, *d* = 0.42
Shallow compensation score (max = 10)	2.76	1.79	0–8	1.81	1.21	0–5	*t*(99.91) = −3.34, *p* = .001, *d* = 0.62
Deep compensation score (max = 9)	1.62	1.45	0–5	1.29	1.02	0–4	*t*(102.11*)* = −1.43, *p* = .16, *d* = 0.27
Masking score (max = 6)	1.53	1.11	0–4	1.61	1.11	0–4	*t*(115) = 0.37, *p* = .71, *d* = 0.07
Accommodation score (max = 6)	0.90	0.85	0–3	0.85	0.93	0–3	*t*(115) = − 0.30, *p* = .77, *d* = 0.06

Greater scores index more self-reported strategies. Effect sizes are reported as Cohen’s *d* (0.2 = small, 0.5 = medium, 0.8 = large). Analyses were conducted using raw and standardised strategy scores (see the “Methods” section). A similar pattern of results was found; therefore, analyses using raw scores are reported (see Additional file 1 for analyses using standardised scores)

Given the inter-relationships between education level, AQ10 and diagnostic status, we sought to investigate which variable was likely driving differences in compensation scores between Diagnosed and Non-diagnosed groups. Therefore, multiple linear regression was used to determine each of their unique contributions to overall and shallow compensation scores, whilst accounting for the other two variables (Table 5). Data were suitable for multiple linear regression as VIF values indicated that multicollinearity was not a concern (all < 10), residuals were normally distributed and Durbin-Watson statistics were ~ 2, suggesting that errors were uncorrelated and thus independent. Overall, education level uniquely and positively predicted overall compensation and both education level and AQ10 uniquely and positively predicted shallow compensation. Notably, having an autism diagnosis was not associated with overall or shallow compensation scores after accounting for AQ10 and education level. Equivalent regression analyses with the other strategy types were not conducted as these variables showed no significant relationship with AQ10 or diagnostic status.

**Table 5:** Regression analysis for overall and shallow compensation scores

Predictor	*β*	*t*	*p*
Overall compensation: *F*(3, 113) = 4.68, *R*^2^ = 0.11, *p* = .004			
Diagnosis (1 = diagnosed, 0 = non-diagnosed)	.11	1.03	.31
Autistic traits	.16	1.45	.15
Highest education level	.20	2.26	.026
Shallow compensation: *F*(3, 113) = 10.08, *R*^2^ = 0.21, *p* < .001			
Diagnosis (1 = diagnosed, 0 = non-diagnosed)	.11	1.10	.28
Autistic traits	.31	2.96	.004
Highest education level	.21	2.43	.017

*β* standardised regression coefficient, *t* Student’s *t* statistic, *p p* value

## Discussion

This study aimed to quantify compensatory strategies in adults with and without autism for the first time. Using the novel 31-item Compensation Checklist, we coded qualitative reports of compensatory strategies used in social situations, to create quantitative compensation scores. We subsequently explored relationships between compensation scores and theoretical correlates of compensation, including diagnostic status, autistic traits, highest education level, age at diagnosis and sex.

Participants reported multiple different strategies. These ranged from masking (i.e. strategies that involve increasing/dampening pre-existing social behaviours and thus ‘hide’ autistic characteristics fairly superficially) to strategies that enable one to appear relatively socially skilled during social interaction, either by circumventing social cognition and using learned ‘rules’ instead (i.e. shallow compensation) or actually finding an alternative way to emulate good social-cognitive ability (i.e. deep compensation). Additionally, we quantified accommodation strategies, which enable one’s autistic behaviours to be accommodated for (e.g. working in an ‘autism friendly’ workplace) and can often work alongside compensation. That these four strategy types were moderately positively correlated suggests separable but overlapping strategies. This corroborates previous research, including the related masking and compensation components of the CAT-Q [15]. This finding also provides novel insights into the wide range of strategies that exist. For example, regardless of diagnostic status, participants tended to report strategies across multiple types, rather than from one strategy type only.

Greater overall compensation scores were associated with greater autistic traits and having an autism diagnosis. This suggests that people may attempt to use compensatory strategies because they genuinely have greater social difficulties to compensate for. That the link with diagnosed autism was found for shallow compensation in particular, supports the idea that shallow compensation strategies may not always be sophisticated enough to disguise autistic tendencies from others, such as clinicians. Additionally, overall and shallow compensation scores were positively linked with education level. This may be due to the fact that compensatory strategies demand intellectual abilities, for example, to work out rules and ‘appropriate’ social behaviours during interaction, when intuitive social understanding is limited [1, 3, 23]. It seems unlikely that this finding was due to people with a higher education level generally having greater self-insight, as education level did not correlate with all strategy types. Additionally, although education level is only an approximation of IQ, this finding corroborates previous findings of a positive link between compensation and IQ test performance [3, 23]. Further, it adds nuance to this literature by suggesting that IQ/education level is in part linked to how *many* compensatory strategies individuals use, i.e. the size of their compensation repertoire. Indeed, higher IQ/education level may aid learning and implementation of multiple strategies, and flexible switching between them.

Notably, however, diagnostic status was no longer associated with compensation scores after accounting for autistic traits and education level. This novel finding indicates that it is more autistic traits (or insight into these), rather than a feature of diagnosable autism (e.g. knowing that you have a diagnosis that makes you different from others), that is linked with greater compensation. The AQ10 is likely picking up social-cognitive difficulties that need to be compensated for; however, it is possible that higher self-report AQ10 scores reflect a greater degree of feeling ‘different from the norm’, which in turn, is associated with the tendency to compensate for this perceived difference. Notwithstanding these various interpretations, there is now clearer evidence that compensation is not limited to clinically diagnosed individuals and it is not diagnosis *per se* that prompts compensatory strategies. This accords with qualitative studies in which autistic adults report using strategies from a young age, before recognition and diagnosis of ASD [5, 7].

Not all strategy types were linked with autism. Masking was not associated with autism diagnosis or autistic traits, which is in line with evidence that non-autistic people also mask certain behaviours for reputation management [5, 7, 15]. Similarly, accommodation and deep compensation strategies were unrelated to both autistic traits and autism diagnostic status. The former finding may be because, like masking, accommodation is not an autism-specific tendency, or instead, that Non-diagnosed individuals are equally likely to use accommodation strategies, potentially contributing to why they have not required an ASD diagnosis. Additionally, we speculate that the latter finding may be because Diagnosed individuals have few deep compensation strategies, which may be indicative of why they required a diagnosis in the first place. Equally, self-reported approaches may not be ideal for studying deep compensation, which can operate without awareness (see Table 1 [5]). Neuro-imaging and neuro-stimulation of non-social neural systems associated with good social-cognitive ability could be more effective methods to study deep compensation in ASD in the future [24].

Unexpectedly, compensation scores were not associated with age at diagnosis, suggesting that compensatory strategies may not necessarily be linked with delayed autism diagnosis, as previously indicated [5–9]. This may in part be because shallow compensation, which was shown in this study to correlate most strongly with autism, can actually be more readily detected by clinicians than deeper compensatory strategies, and therefore, shallow compensation is less likely to contribute to delayed diagnosis. Further research using other compensation measures is now required, for example, behaviour-cognition discrepancy approaches [2, 3] and brain imaging of unconscious cognitive processes which better capture deep compensation [24]. This research should use a broader range of diagnosis age than our sample, in which 48/58 were diagnosed in adulthood, and consider compensation alongside other factors associated with delayed diagnosis (e.g. lower socioeconomic status [25]). Further, there was no association between compensation scores and sex in our study, suggesting that males and females use compensatory strategies to similar degrees, although the number of males in sample was small (*n* = 22). This speaks against the notion that the female autism phenotype is characterised by high levels of compensation [1, 2], and instead fits with mounting evidence that autistic males also engage in compensation [3, 5, 7, 15, 26], although there may be sex-specific reasons for compensation [16].

Our findings have crucial implications for research and clinical practice. We suggest that clinicians should be aware of compensatory strategies in intellectually able individuals reporting autistic-like difficulties, even if they do not meet strict behavioural criteria for ASD. Indeed, these individuals may require a similar level of support to diagnosed individuals, particularly as compensation is linked with poor mental wellbeing [1–3, 5, 7, 15, 16]. Further, measuring self-reported compensation in clinical settings (e.g. using the Compensation Checklist) may help to detect autistic tendencies in ‘well-compensated’ individuals whose condition is hidden in behaviour. Indeed, the Diagnostic and Statistical Manual for Mental Disorders [27] now acknowledges that strategies may disguise clear-cut autistic behaviours, and our checklist offers a first step for clinicians to begin measuring these strategies. Such tools could supplement traditional observational diagnostic processes, to give insight into individuals’ (hidden) social difficulties and improve diagnostic precision [28].

### Limitations

There are several limitations and promising directions for future research. First, it remains unclear whether the self-reported compensatory strategies captured by the Compensation Checklist necessarily translate into neurotypical social behaviour, as we did not measure strategy frequency or success. Future research should assess self-reported compensatory strategies alongside observer-rated measures of social behaviour. Second, we used a convenience sample and therefore replication is required in larger and more representative (e.g. population-based) samples, including individuals with subtler forms of ASD and equal numbers of males and females [29]. In particular, we were potentially under-powered to detect sex differences, given the small number of males in the sample, although it is noteworthy that effect sizes were also small. Third, given the self-report nature of the study, our results, alongside most research findings on compensation in ASD so far, are not necessarily representative of autistic people with additional intellectual disability. Moving forward, observational and carer-report methods may be required to assess compensatory strategies in autistic individuals who are less able to verbally report such strategies. Finally, we note that there was low internal consistency of the individual strategy subtypes, but good internal consistency of the Compensation Checklist as a whole. Indeed, there may be conceptually similar strategies that cannot practically operate together at the same time. Moving forward, we suggest that the Compensation Checklist is used in full, and caution against the measurement of subtypes in and of themselves, until these subtypes are further validated.

## Conclusions

Overall, the Compensation Checklist may be a useful tool for quantifying compensatory strategies in adults with and without autism. It is likely to have better utility in time-limited research and clinical sessions, compared with lengthy cognitive and behavioural tasks. Our findings build upon previous literature suggesting that compensatory ability is closely related to intellectual ability and self-reported compensatory strategies are not limited to individuals with diagnosed autism. Our findings, however, did not confirm the expected relationship between self-reported compensation and age at diagnosis and female sex, although further high-powered research is required. We suggest that the Compensation Checklist offers a first step for clinicians seeking methods to measure compensatory strategies during autism assessments. We envisage it be used as a prompt for clinicians to directly ask questions about compensatory strategies during autism assessments, or rephrased and validated as a self- or carer-report measure. The efficacy of the tool for improving diagnostic accuracy and clinical support for autistic people will require thorough investigation.

## Supplementary information

**Additional file 1.** Supplementary materials.

## Data Availability

The anonymised data from the present study are available from the corresponding author on reasonable request.

## Abbreviations

AQ10: 10-item Autism-Spectrum Quotient
ASD: Autism spectrum disorder
IQ: Intelligence quotient

## Footnotes

1 Gwet’s AC1 [20] was the only appropriate measure of inter-rater reliability as, unlike other measures (e.g. Cohen’s kappa), it is robust against a skew in reliability due to unequal distribution of binary responses (see [21]).
