# Supplementary material for: Quantifying compensatory strategies in adults with and without diagnosed autism
Source: Mol Autism. 2020 Feb 12;11:15. doi: 10.1186/s13229-019-0308-y (PMC7014718; doi:10.1186/s13229-019-0308-y)
Supplement: Supplementary file 1 — Additional file 1. Supplementary materials. [file 13229_2019_308_MOESM1_ESM.docx]

**Supplementary Materials**

The 31-item *Compensation Checklist* used to code qualitative responses is shown in Appendix 1.

**Results**

Equivalent analyses to those reported in the Main Text were conducted using standardised scores for overall compensation, masking, shallow compensation, deep compensation and accommodation (see Main Text Methods for detail about standardisation procedure). A similar pattern of results was found using standardised scores (see Supplementary Tables 1-4).

**Supplementary Table 1.** Correlational analyses using standardised strategy scores.

|  | 1 | 2 | 3 | 4 | 5 |
| --- | --- | --- | --- | --- | --- |
| Overall Compensation (1) | - | .64*** | .54*** | .64*** | .63*** |
| Shallow Compensation (2) |  | - | .13 | .16 | .28** |
| Deep Compensation (3) |  |  | - | .13 | .18 |
| Masking (4) |  |  |  | - | .15 |
| Accommodation (5) |  |  |  |  | - |
| Autistic Traits | .22** | .41*** | .01 | .07 | .05 |
| Highest Education Level | .22* | .25** | .02 | .09 | .18* |
| Sex (1 = Female, 0 = Male)^a^ | -.03 | -.11 | .03 | .07 | -.10 |
| Diagnosis (1 = Diagnosed, 0 = Non-diagnosed)^a^ | .16* | .30** | .13 | -.03 | .03 |
| Age at Diagnosis^b^ | .15 | .04 | -.08 | .19 | .22 |

*Note*. Highest education level was used as a proxy IQ measure. Greater scores reflect higher education level/greater autistic traits/more self-reported strategies. **p* < .05 ***p* < .01 ****p* < .001. ^a^Point-biserial correlations. ^b^Diagnosed group only (*n* = 58).

**Supplementary Table 2.** Group-wise comparison of standardised strategy scores.

|  | **Diagnosed (*n* = 58)**  *M*             *SD*          Range | **Non-diagnosed (*n* = 59)**  *M*            *SD*          Range | Comparison |
| --- | --- | --- | --- |
| Overall Score | 0.86         0.43 0.11-1.98 | 0.73         0.34 0.10-1.68 | *t*(115) = -1.78 *p* = .077 *d* = 0.33 |
| Shallow Compensation Score | 0.28         1.78 0-0.80 | 0.18         1.21 0-0.50 | *t*(99.91) = -3.34 *p* = .001 *d =* 0.62 |
| Deep Compensation Score | 0.18         0.16 0-0.56 | 0.14         0.11 0-0.44 | *t*(102.112*)* = -1.43 *p* = .16 *d* = 0.27 |
| Masking Score | 0.26         0.19 0-0.67 | 0.27         0.19 0-0.67 | *t*(115) = 0.37 *p* = .71 *d* = 0.07 |
| Accommodation Score | 0.15         0.14 0-0.50 | 0.14         0.15 0-0.50 | *t*(115) = -0.30 *p* = .77 *d* = 0.06 |

*Note*. Greater scores index more self-reported strategies. Effect sizes are reported as Cohen’s *d* (0.2 = small, 0.5 = medium, 0.8 = large).

**Supplementary Table 3.** Regression analysis for standardised overall and shallow compensation scores.

| **Overall Compensation:** *F*(3, 113) = 3.64, *R*^2^ = 0.09, *p* = .015 | | | |
| --- | --- | --- | --- |
| **Predictor** | ***β*** | ***t*** | ***p*** |
| Diagnosis (1 = Diagnosed, 0 = Non-Diagnosed) | .08 | 0.70 | .48 |
| Autistic Traits | .15 | 1.29 | .20 |
| Highest Education Level | .20 | 2.19 | .030 |
| **Shallow Compensation:** *F*(3, 113) = 10.08, *R*^2^ = 0.21, *p* < .001 | | | |
| Diagnosis (1 = Diagnosed, 0 = Non-Diagnosed) | .11 | 1.10 | .28 |
| Autistic Traits | .31 | 2.96 | .004 |
| Highest Education Level | .21 | 2.43 | .017 |

*Note*: *β* = Standardised regression coefficient, *t* = Student’s t-statistic, *p* = p-value. Examination of VIF values across the regression analyses indicated that multicollinearity was not a concern (all <10), and the residuals were normally distributed. Durbin-Watson statistics were inspected and found to be ~2 across the regression analyses, suggesting that errors were uncorrelated and thus independent. Together, the data were suitable for multiple linear regression analysis.

**Appendix 1 – Compensation Checklist**

| **Strategy Name** | **Description** | **Present (1) Absent (0)** |
| --- | --- | --- |
| **Masking** | | |
| 1. Avoidance | Avoid social situations where you would stand out. |  |
| 1. Hold back | Hold back your true thoughts and opinions in conversation (e.g., agree with others even if you disagree with them, tolerate behaviour of others). Hide aspects of your personality that would be deemed different to the norm (e.g., your interests and hobbies). |  |
| 1. Suppress | Suppress atypical behaviours (e.g., hand flapping, fidgeting). |  |
| 1. Present but passive | Attend social events, even if you would rather not, to give the impression of sociability. Stand in a conversation but say/do as little as possible. |  |
| 1. Superficial assimilation | Dress and speak like the group you are trying to blend in with (e.g., copy hairstyle, language, interests). |  |
| 1. Basic social etiquette | Reflect basic social etiquette to indicate a willingness to socialise (e.g., smile, manners, look towards other people). |  |
| **Shallow Compensation** | | |
| 1. Plan and rehearse | Predict, plan out and rehearse conversations before they happen, out loud or in your head. |  |
| 1. Copy/model behaviour | Mimic phrases, gestures, facial expressions, tone of voice picked up from other people and/or TV/film/book characters. |  |
| 1. Eye contact | Make appropriate eye contact, even if it is not useful for communication and/or is aversive. Avoid eye contact but give the impression of social interest (e.g., look at bridge of nose, stand at a 90° angle to interaction partner). |  |
| 1. Learned scripts, social rules | Enact learned scripts and social rules, even when it may not be appropriate, to guide conversations (e.g., ask others set questions, small talk, laugh at ‘joke cues’, turn-take in conversation). |  |
| 1. Guide conversation | Steer conversation to topics you are equipped to talk about (e.g., special interests). Focus conversation on your interaction partner to draw attention away from self. |  |
| 1. Rely on others | Attend social events with a more socially skilled individual who can act as a ‘social crutch’ (e.g., introduce you, fill in or disguise your social mistakes, explain social nuances to you). |  |
| 1. Reduce social demands | Reduce social demands on yourself in order to disguise any social faux pas (e.g., ‘flit’ between different groups/conversations, engage in 1:1 conversation rather than groups so there are less social signals to read, make use of structured socialising or ‘organised fun’). |  |
| 1. Counselling skills | Listen to, repeat and rephrase what your interaction partner says to give the impression of being an ‘good listener’ or ‘adviser’, without having to necessarily mentalise. |  |
| 1. Use props | Rely on props (e.g., dog, children, interesting object) to structure and guide conversation. Similar to learned scripts. |  |
| 1. Play a false role | Play an exaggerated role or character that is inconsistent with the ‘real you’ (e.g., false confidence, fabricated stories, extraverted personality). |  |
| **Deep Compensation** | | |
| 1. Learned non-verbal cues | Use learned rules about non-verbal behaviour (e.g., facial expression, body language, direction of gaze), when it is appropriate, to infer what others are thinking/feeling. For example, inferring that when someone looks at the ground or rolls their eyes, they are bored. |  |
| 1. Learned verbal cues | Use learned rules about verbal behaviour (e.g., tone of voice, content of speech) to infer what others are thinking/feeling. For example, inferring that someone who is talking about a funeral with a particular tone is likely sad. |  |
| 1. Assess behaviour | Assess someone’s behaviour over time to infer what they are thinking/feeling. For example, if someone re-invites you to a social event, they think positively of you. |  |
| 1. Substituted perspective taking | Substitute someone else’s values/preferences/interests with your own or those of a TV/film/book character to infer what others are thinking/feeling. For example, if someone is acting similar to a TV/film/book character in particular situation, infer that they are thinking/feeling how that character would in the same situation. |  |
| 1. Logic, context, experience | Predict likelihood of what someone is thinking/feeling based on logic, the context or experience of how that person has previously behaved. Often involves analysing social situations after they have happened and carrying ‘lessons learned’ to the next time the same situation happens. |  |
| 1. Flexible catalogue | Flexibly use built catalogue of possible interpretation of others’ thoughts/feelings, based on combination of multiple sources of information (e.g., logic, context, facial expression, tone of voice). |  |
| 1. More information or time | Gain more information to increase accuracy of your inference about someone’s thoughts/feelings (e.g., get them to repeat what they have said in a different way, find out about their interests/opinions from others). Gain more time to make a judgement of someone’s thoughts/feelings (e.g., take a well-timed break to consider various interpretations). |  |
| 1. Recalibrate | Recalibrate your interpretation of someone else’s thoughts/feelings based on self-awareness of your own cognitive biases (e.g., tendency to perceive neutral expression as anger). |  |
| 1. Psychological theory | Apply learned psychological theory to help infer what others are thinking/feeling (e.g., categorise people by personality type). |  |
| **Accommodation** | | |
| 1. Play to your strengths | Play to your strengths (e.g., humour, wit, intelligence) to add additional value to conversation with others, despite your social differences. |  |
| 1. Be helpful, liked | Go out of your way to be helpful to others, so that your social differences might be forgiven. |  |
| 1. Seek ‘atypical’ others | Seek relationships with others who are also socially ‘atypical’ and therefore more accepting of your social differences. |  |
| 1. Accommodating environment | Work in an environment where your social differences are actively accommodated (e.g., ‘autism friendly’ workplace) or where non-social skills are valued over social ones (e.g., academia, skill-based job). |  |
| 1. Foreign disguise | Live in a foreign country so that your differences are attributed to being foreign by others. Live in your country of birth but seek relationships with others who are foreign, so that your social differences are attributed to cultural differences. |  |
| 1. Disclose difficulties | Disclose your difficulties or diagnosis to others so that they can better accommodate you. |  |
